# Exploring the feasibility of AI-based analysis of histopathological variability in salivary gland tumours

**DOI:** 10.1038/s41598-025-15249-5

**Published:** 2025-08-09

**Authors:** Ibrahim Alsanie, Adam Shephard, Neda Azarmehr, Pablo Vargas, Miranda Pring, Nasir M. Rajpoot, Syed Ali Khurram

**Affiliations:** 1 Department of Oral Medicine and Diagnostic Sciences, College of Dentistry, King Saud University, Riyadh, Kingdom of Saudi Arabia; 2 Tissue Image Analytics Centre, Department of Computer Science, University of Warwick, Coventry, UK; 3 School of Information, Journalism and Communication, University of Sheffield, Sheffield, S10 2AH UK; 4 Oral Diagnosis Department, Piracicaba Dental School, University of Campinas (UNICAMP), Piracicaba, São Paulo, Brazil; 5 Bristol Dental School, University of Bristol, Bristol, BS2 OPT UK; 6 School of Clinical Dentistry, Faculty of Health, University of Sheffield, Claremont Crescent, Sheffield, S10 2TA UK

**Keywords:** Salivary gland tumours, Artificial intelligence, Machine learning, Deep learning, Image analysis, Histopathology, Diagnostic markers, Biomarkers

## Abstract

This study uses artificial intelligence (AI) for differentiation between salivary gland tumours (SGT) using digitised Haematoxylin and Eosin stained whole-slide images (WSI). Machine learning (ML) classifiers were developed and tested using 320 scanned WSI. These included a benign versus malignant classifier (BvM) for automated identification of benign and malignant tumours, a malignant sub-typing (MST) classifier for subtyping four most common malignant SGT and a third classifier for malignant tumour grading. ML results were also compared with deep learning models. All ML classifiers showed an excellent accuracy. An F1 score of 0.95 was seen for benign vs. malignant and malignant subtyping tasks and 0.87 for automated grading. In comparison, the best performing DL models showed F1 scores of 0.80, 0.60 and 0.70 for the same tasks respectively. External validation on an independent cohort demonstrated good accuracy, with an F1 score of 0.87 for both the benign vs. malignant and grading classifiers. A notable association between cellularity, nuclear haematoxylin, cytoplasmic eosin, and nucleus/cell ratio (*p* < 0.01) were seen between tumours. Our novel findings show that AI can be used for automated differentiation between SGT. Analysis of larger multicentre cohorts is required to establish the significance and clinical usefulness of these findings.

## Introduction

Salivary gland tumours (SGTs) are a heterogeneous group of neoplasms that present with a wide range of histological features and subtypes. They are rare, with an annual incidence of approximately 2.5–3.0 per 100,000 people in the Western world^1^. SGTs can be diagnostically challenging due to a large number of entities and markedly similar and overlapping features but different clinical behaviour^2^. A number of additional special stains, immunohistochemistry (IHC), and molecular work (Fluorescence in Situ Hybridisation (FISH), Polymerase Chain Reaction (PCR), etc.) can be required to diagnose and sub-type SGT. However, these tests may only be available at specialist centres with associated time delays and cost implications. In some cases, even when these investigations can be performed, differentiation between tumour types can be challenging in particular on small biopsies^3^, and unfortunately, very few of these special tests are available in the developing world.

Furthermore, contextual and morphological information is not evident when molecular or genetic tests are performed, and a number of SGTs have no known molecular alterations at present. Identification of the tumour type is important as it provides guidance for the optimal management and prognostic behaviour. These issues highlight the need for novel, efficient, and widely available methods as diagnostic aids.

The advent of digital pathology and the ability to obtain whole slide images (WSIs) from histology slides has accelerated the application of artificial intelligence (AI) to pathology, which has been helped by advancements in computing power and technology, allowing exploration of sub-visual morphometric features with a potential to improve patient care^4^. Numerous reports have shown the use of AI for consistent and quantitative histological diagnosis as well as providing prognostic information in a range of cancers^5–10^. However, its application to SGTs has not been investigated or reported to date.

Machine learning (ML) is a branch of AI that learns from data that has been provided to make a prediction^11–13^. ML techniques have been employed by several image analysis platforms to analyse cell, tissue and histology images. Open-source image analysis platforms with built-in ML capabilities (such as QuPath) have been utilised for histological feature analysis and staining quantification^14^.

Deep learning (DL) encompasses a range of approaches that are based on ANNs, but typically with many hidden layers. Convolutional neural networks (CNNs) are a DL approach that has become dominant in the computer vision and computational pathology literature for both segmentation and classification tasks, owing to their efficiency on inference and generalisability to new data^4^.

Recent efforts have applied deep learning models to SGT, focusing primarily on subtype classification^15,16^. Additional studies have addressed specific rare histologic entities^17^, immune-mediated conditions such as Sjogren’s syndrome^18^. However, most prior work has not tackled the combined diagnostic challenges of benign vs. malignant differentiation, malignant subtyping, and histological grading, which are critical for clinical decision-making. Our study aims to address these three tasks within a real-world dataset using interpretable ML and DL tools. For this purpose, QuPath was used for feature generation within manually selected regions of interest (ROIs). The ROIs were then exported to create the ML classifier using established Python libraries. Finally, owing to the recent popularity and success of DL techniques, these results were compared with DL models applied to the same tasks.

## Materials and methods

Benign and malignant SGTs were identified using a digital database and the corresponding Haematoxylin & Eosin (H&E) stained slides were retrieved from the department archive (Ethics reference: 20/WS/0017). This department receives a large number of regional, national and international consult cases for expert opinion, and both internally and externally stained slides were included in the cohort to ensure the robustness of training. The retrieved cases included two common benign SGT subtypes, i.e., pleomorphic adenoma (PA) and basal cell adenoma (BCA), and four malignant SGT subtypes, i.e., mucoepidermoid carcinoma (MEC), adenoid cystic carcinoma (AdCC), acinic cell carcinoma (ACC) and carcinoma ex pleomorphic adenoma (Ca-ex-PA). All cases were independently reviewed and confirmed by two oral and maxillofacial pathologists (IA and SAK) prior to slide digitization. Consensus diagnosis was established through joint review when discrepancies arose. Ancillary tests such as IHC were considered in selected cases where additional diagnostic clarification was required, followed by anonymisation of the slides. Whole slide images (WSIs) were generated using Aperio CS2 and Hamamatsu Nano-zoomer scanners at 40x magnification. Calibration was done prior to each scanning session, and images were stored on a dedicated server. Anonymised WSIs of cases to be analysed were downloaded from the server for analysis.

A wide range of features was analysed and compared between tumours, including cellularity, nuclear and cytoplasmic features such as circularity, eccentricity, haematoxylin and eosin (H&E) optical density and nucleus/cell area ratio.

### Case selection and building of machine learning classifiers

For comparison between benign and malignant SGT, WSIs of H&E-stained sections were used, including 120 from benign tumours (PA and BCA) and 120 from malignant tumours (including MEC, AdCC, ACC, and Ca-ex-PA). We used 67% (*n* = 160) of the cases for training and the remaining for testing (*n* = 80) (Table 1). In our cohort, approximately 75% of benign tumours originated from major salivary glands and 25% from minor glands. In contrast, malignant tumours demonstrated an even distribution, with 50% arising in major glands and 50% in minor salivary gland sites. An open-source bioimage analysis software (QuPath) was employed initially for annotation, feature generation and extraction of ROIs^14^. To overcome varying staining procedures between laboratories, Ruifrok and Johnston’s colour deconvolution technique is utilised by QuPath, which allowed reliable separation of haematoxylin and eosin signals for downstream analysis^19^.

**Table 1:** Training and testing sets case numbers breakdown for benign vs. malignant (BvM), malignant tumour subtyping (MST) and tumour grading (TG) classifiers.

Classifier type	Training set	Test set	Total
Benign vs. malignant (BvM)	160 WSIs	Local (80 WSIs) + External (40 WSIs) = 120 WSIs	280 WSIs
Malignant tumour subtyping (MST)	80 WSIs	40 WSIs	120 WSIs
Tumour grading (TG)	80 WSIs	Local (40 WSIs) + External (40 WSIs) = 80 WSIs	160 WSIs

**Fig. 1:**
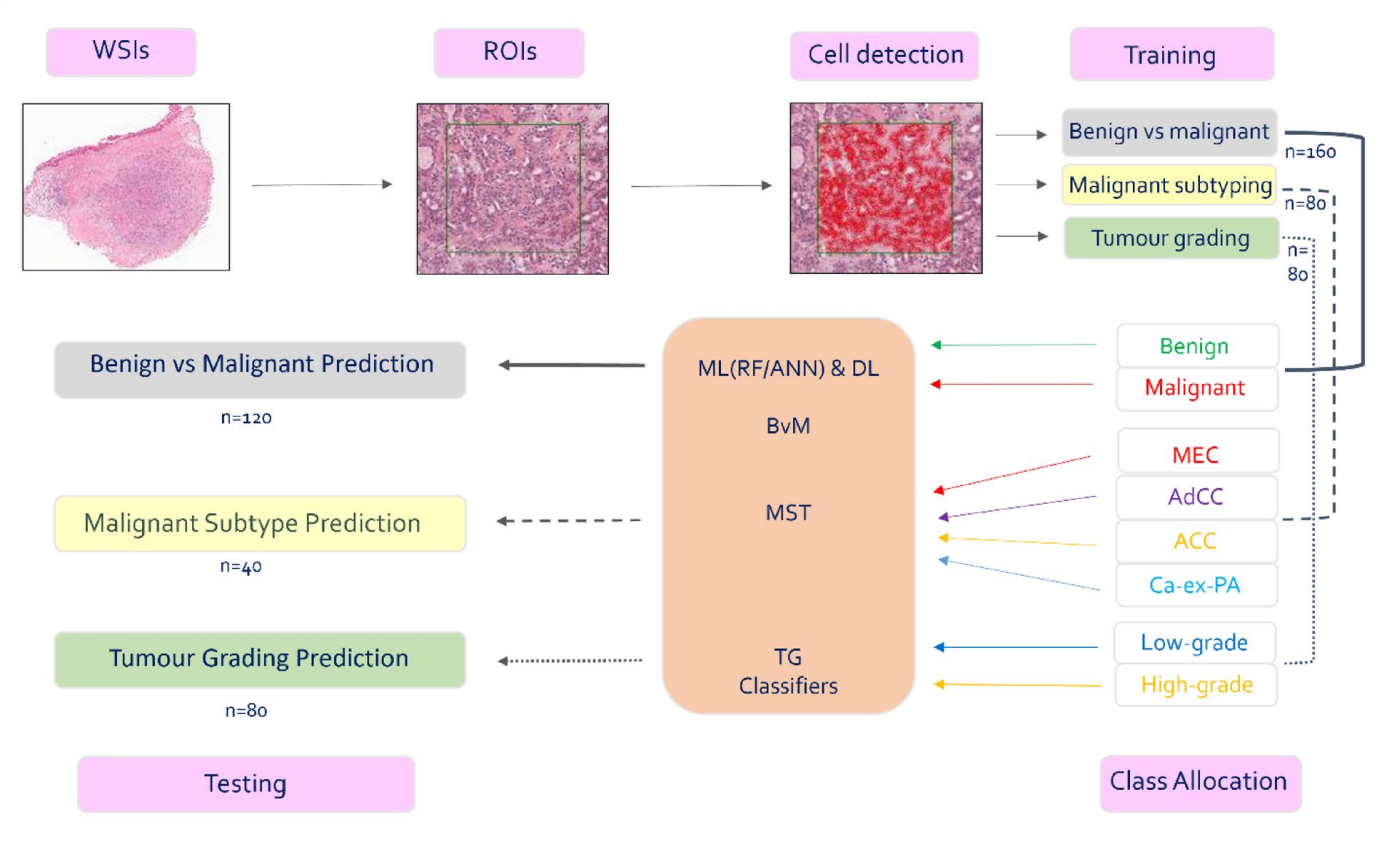
Training and analysis overview.

To train the benign vs malignant (BvM) detection classifier, ROIs per WSI were selected using fixed-size areas of 142,884 µm^2^ (1500 × 1500 pixels) to ensure standardisation across cases. Next, cell detection analysis was performed, following which the detected cells/nuclei were assigned to a specific class/ground truth. To address intra-tumoral heterogeneity, a minimum of five morphologically distinct ROIs were manually selected from each WSI for training. These ROIs captured representative variations in tumour architecture and cytology, including patterns such as cribriform, clear cell, solid, and tubular areas when present (where applicable). This approach aimed to ensure the model’s exposure to the full spectrum of histologic features within each tumour type. Using the ROIs in the training cases, a ML classifier (Random Forest/RF) was built and validated through visualisation of nuclear segmentation in the unseen cases using the *Scikit-learn 1.0.1* toolbox with Python^20^. 80 unseen WSIs with 400 ROIs of fixed-size areas were used to blindly test and validate the BvM classifier for automated classification, followed by quantification and statistical analysis of colour and morphometric features that were contributing to the classifier’s ‘decision’ (Fig. 1) (Supplementary Table 1). In addition to the local test set, external validation was performed using 40 unseen WSIs of benign and malignant salivary gland tumours (SGTs), including 20 benign cases (PA) from the Piracicaba Dental School, University of Campinas (Brazil), and 20 malignant cases (10 MEC and 10 AdCC) from the Head and Neck 5000 cohort^21^ (http://www.headandneck5000.org.uk/) (REC reference: 10/H0107/57).

The second part of the study aimed to build an additional classifier for malignant tumour subtyping (MST) for automated identification of the most common malignant SGT. 120 WSIs were used for training and testing. Although these cases were the same as those used in the previous part of the study, a new classifier was built to ensure that the test cases remained ‘unseen’. Two-thirds (*n* = 80) WSIs were used for training and the remainder for testing (*n* = 40) (Table 1). A RF classifier was trained using 80 WSIs with 400 ROIs, including four different tumours (MEC, AdCC, ACC, and Ca-ex-PA) (*n* = 20 WSIs of each tumour). The ROI dimensions were maintained at 1500 × 1500 pixels, and cell/nuclear detection was performed as described previously. All detected cells in each tumour type ROI were assigned to a specific tumour type class (i.e., MEC, AdCC, ACC, Ca-ex-PA). 40 unseen WSIs (10 of each SGT) with 200 ROIs were used to test the MST classifier and to perform analysis and quantification of features (Fig. 1). External validation of the MST classifier was not feasible due to the limited representation of distinct malignant SGT subtypes within the available external cohort. Including such a restricted dataset could have introduced a significant class imbalance, potentially compromising the reliability and generalizability of the validation results.

The third part of the study intended to build a tumour grading classifier (TG) for the most two common gradable SGT MEC and AdCC. Two-thirds of cases (80 WSIs) were used for training and unseen one-third for testing (internal set (*n* = 40)), an additional external dataset comprising 40 cases from the Head and Neck 5000 cohort was used for validation. This included 20 MEC and 20 AdCC (10 low-grade and 10 high-grade for each) (Table 1). Due to subjectivity of the three-grade system in interpreting histological features a binary grading system of low or high grade was used for both tumours. The grading criteria were consistent with existing literature. The grading of MEC is based on the most prominent histological features that can be seen in the tumour, with low-grade MEC being defined by a predominance of mucous cells, a cystic or glandular growth pattern, and showing a low mitotic rate and minimal cellular pleomorphism, while high-grade tumours are characterised by a predominance of epidermoid cells, a lack or minimal mucous cells, a solid or infiltrative growth pattern, and marked cellular pleomorphism. The intermediate grade was joined with the nearest grade according to above mentioned criteria (based on the Brandwein and AFIP score). In addition, the grading of AdCC is based on the predominant pattern observed in the tumour, with the cribriform and tubular patterns were considered as low-grade and solid patterns as well as cases that demonstrated necrosis as high-grade (any tumours with solid areas were therefore categorised as high grade). A binary grading system of low or high grade was used. The classifiers were trained by selecting larger 3–5 ROIs per WSI using fixed-size areas of 1,587,600 µm^2^ (5000 × 5000 pixels). ML artificial neural network (ANN-ML classifier) was built and validated through the visualisation of nuclear segmentation. Finally, an automated analysis of features was performed (Fig. 1).

### Comparison with deep learning networks

Next, the utility of DL methods for classification was analysed. For direct prediction with DL, the ROIs were tessellated into smaller patches (256 × 256, at 40×) before training/testing multiple state-of-the-art convolutional neural networks (CNNs) for automated prediction. Here, we used ResNet-18, ResNet-50, Efficient-NetB0 and Efficient-NetB3 models, built with PyTorch 1.10 ^22,23^. On inference, the maximum argument was taken across all patches per subject to achieve predictions.

In this work, we have therefore performed two different sets of experiments:

1. Feature generation and employing ML (RF and ANN) for classification.
2. DL for classification (based on raw image patches).

### Spatial analysis

The spatial analysis was performed using a set of features related to the orientation of objects at a certain location. This included proximity by measuring centroid distance between cells as well as cluster Delaunay analysis, including neighbouring cells, intercellular distance, and mean triangle area. Delaunay triangulation is a geometric calculation indicating a set of points that can be found in optimal time and position^24^.

### Statistical analysis

T-Test and multiple comparison one-way ANOVA were used to evaluate differences across various geometrical, spatial, and staining features. Microsoft Excel 2016 (Microsoft Office Software, USA) was used to organise exported data and perform statistical analyses. The performance of detection classifiers was measured using precision, recall, F1 score, and AUROC, generated at the case level.

## Results

### Performance of the classifiers

**Fig. 2:**
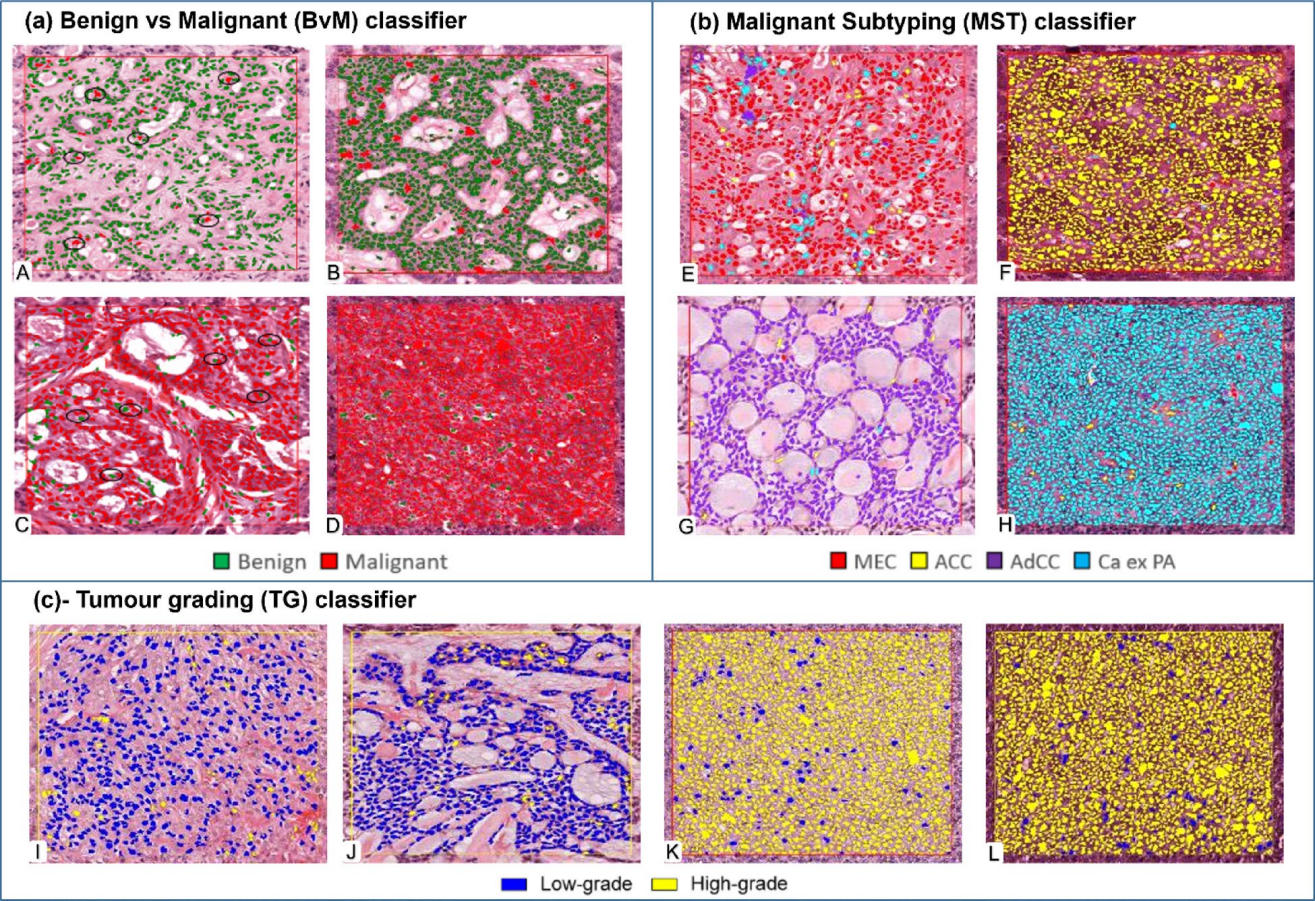
Automatic cell segmentation and classification in ROIs using trained classifiers. (**a**) (BvM, test *n* = 80) predictions were largely correct with some false-positive and false-negative cells (highlighted by black circle in A & C). A: Pleomorphic adenoma (benign), B: Basal cell adenoma (benign), C: Mucoepidermoid carcinoma (malignant), D: Acinic cell carcinoma (malignant). (**b**) (MST, test *n* = 40), most of the predicted classes were correct with some false positive detections. E: Mucoepidermoid carcinoma (red), B: Acinic cell carcinoma (yellow), C: Adenoid cystic carcinoma (purple), D: Carcinoma ex pleomorphic adenoma (cyan). (**c**) (TG, test *n* = 40) predictions were largely correct with some false positive cells. I: Mucoepidermoid carcinoma (low-grade), J: Adenoid cystic carcinoma (low-grade), K: Mucoepidermoid carcinoma (high-grade), L: Adenoid cystic carcinoma (high-grade) (magnification ×20).

The benign versus malignant tumour RF classifier (BvM) showed high F1 and AUROC scores of 0.95 (Table 2). The predominant detection/class was accurate for all cases, although some isolated false positive and false negative detections for both benign and malignant cells were identified (Fig. 2). Results from DL were similar, with all CNNs showing high F1-scores (> 0.80, Table 2), with EfficientNet-B0 giving the best performance (F1 = 0.87). However, none of the DL CNNs surpassed the performance achieved using the ML classifier (F1 = 0.95). Testing of the ML classifiers on the external cohort also showed an excellent performance (F1 score = 0.87) (Table 2).

**Table 2:** Performances/accuracy metrics of the different classifiers at case-level predictions.

Classifier	Precision	Recall	F1-score	AUROC
Benign vs. malignant (BVM)				
ML (RF)	0.93	0.98	0.95	0.95
DL (ResNet-18)	0.83	0.80	0.81	0.81
DL (ResNet-50)	0.85	0.87	0.86	0.86
**DL (EfficientNet-B0)**	**0.93**	**0.82**	**0.87**	**0.87**
DL (EfficientNet-B3)	0.88	0.76	0.81	0.81
Malignant subtyping (MST)				
**ML (RF)**	**0.95**	**0.95**	**0.95**	**0.97**
**DL (ResNet-18)**	**0.60**	**0.60**	**0.60**	**0.73**
**DL (ResNet-50)**	**0.63**	**0.61**	**0.60**	**0.74**
DL (EfficientNet-B0)	0.58	0.60	0.56	0.73
DL (EfficientNet-B3)	0.55	0.60	0.54	0.73

Significant values are in bold.

RF, Random Forest. Produced using the scikit-learn Python toolbox.

The malignant subtyping ML classifier (MST) also showed excellent performance for classification and automatic differentiation between SGT subtypes (including MEC, AdCC, ACC and Ca-ex-PA), with the predominant automated detection correct in all instances (F1 = 0.95, AUROC = 0.97, Table 2). However, there were some false-positive detections as shown in Fig. 2. Interestingly, results for DL models appeared inferior to the ML classifier (highest F1 score = 0.60 with ResNet-18 and ResNet-50) (Table 2).

The tumour grading ML classifier (TG) showed high F1 and AUROC scores of 0.87 and 0.89, respectively (Table 2, Fig. 2). The state-of-the-art DL models performed inferior to the ML classifier (highest F1 score = 0.70 with EfficientNet-B0) (Table 2). Validation of the ML classifier on the external cohorts also showed an excellent performance with an F1 score of 0.87 (Table 2).

**Fig. 3:**
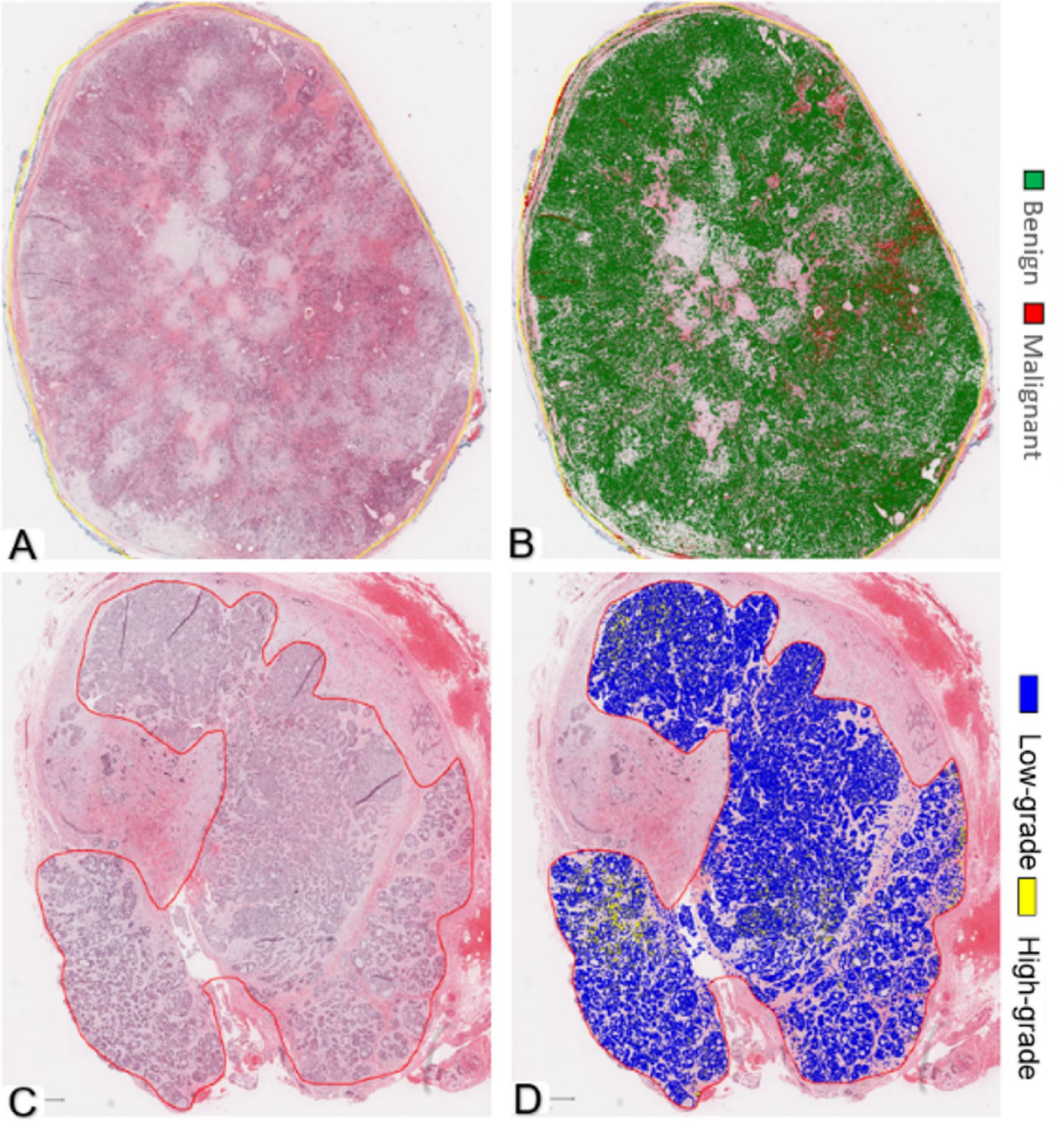
Automatic cell segmentation and classification at WSI level. (**A**,**B**) Using BvM classifier on benign case (pleomorphic adenoma). (**A**) Ground truth of H&E WSI, (**B**) Heatmap prediction. (**C**,**D**) using TG classifier on low-grade tumour case (adenoid cystic carcinoma). (**C**) Ground truth of H&E WSI, (**D**) Heatmap prediction. (magnification ×1).

To enhance model transparency and facilitate clinical interpretability, we incorporated heatmap visualizations of AI predictions at the WSI level. As illustrated in Fig. 3, the BvM and TG classifiers generate spatial probability maps.

For the benign case (PA), the BvM classifier correctly predicted the overall benign diagnosis (Fig. 3A,B). In contrast, the TG classifier applied to a low-grade AdCC case (Fig. 3C,D) highlighted the overall low-grade predication.

### Features contributing to the classifier performance

**Fig. 4:**
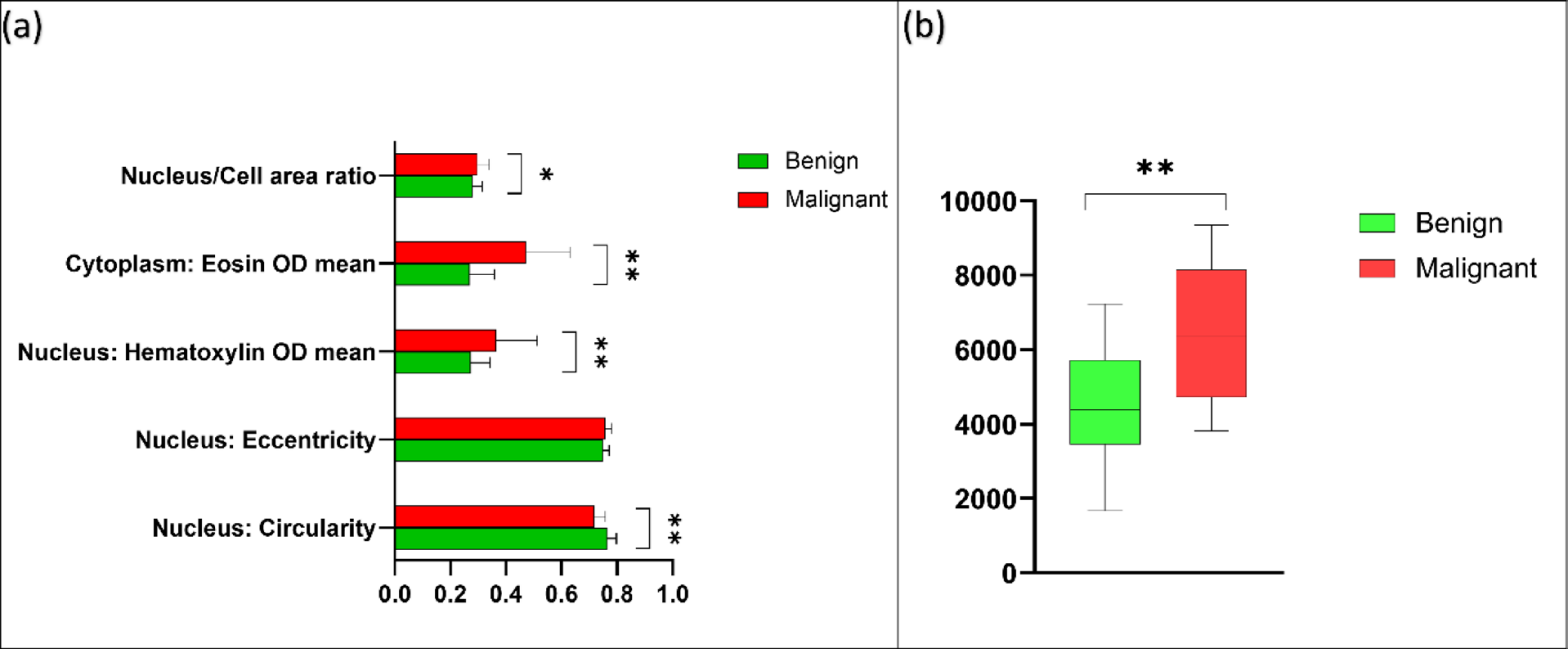
(**a**) Average values for nuclear and cytoplasmic features in benign (green) and malignant (red) unseen test cases (test *n* = 80). (**b**) Boxplot showing cellularity of the benign and malignant tumours (test *n* = 80). Error bars = standard deviation. *p < 0.05, **p < 0.01 (T-Test (two-tailed).

**Fig. 5:**
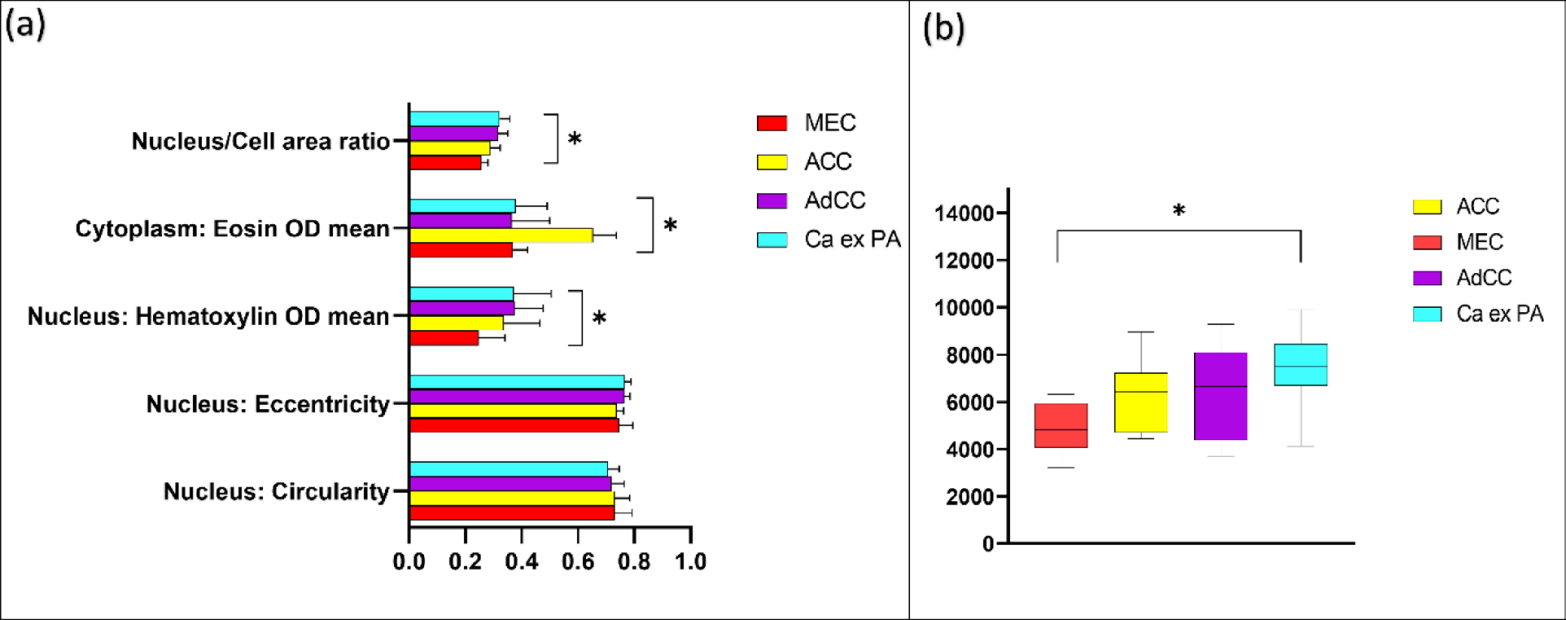
(**a**) Average nuclear and cytoplasmic feature values for the malignant subtyping unseen test set (*n* = 40). Mucoepidermoid carcinoma (red), Acinic cell carcinoma (yellow), Adenoid cystic carcinoma (purple) and Carcinoma ex pleomorphic adenoma (cyan). (**b**) Boxplot showing the cellularity comparison of malignant tumours (test *n* = 40). Error bars = standard deviation. *p < 0.01 (Multiple comparison one-way ANOVA).

**Fig. 6:**
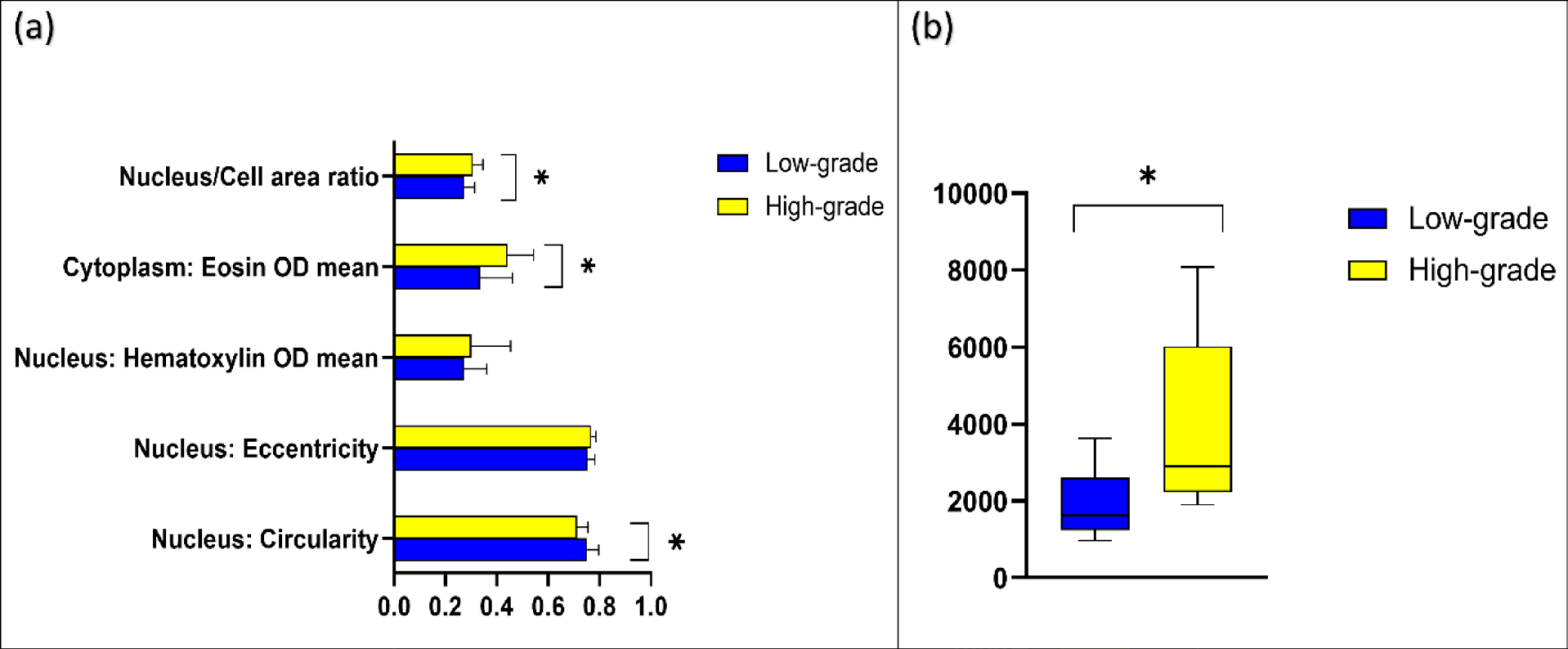
(**a**) Average values for nuclear and cytoplasmic features in low grade (blue) and high grade (yellow) unseen test cases (test *n* = 40). (**b**) Boxplot showing cellularity of the benign and malignant tumours (test *n* = 40). Error bars = standard deviation. *p < 0.01 (T-Test (two-tailed).

The geometrical and morphometrical feature analysis of the test set demonstrated a number of features that were being used by the BvM classifier for benign or malignant class prediction. Analysis indicated a clear variation between nuclear circularity, nuclear haematoxylin optical density (OD), and cytoplasmic eosin OD between benign and malignant tumours (*p* < 0.01). Furthermore, a notable difference in nucleus/cell ratio was also seen (*p* < 0.05). No difference was seen between benign and malignant SGT for nuclear eccentricity (Fig. 4a). Similar to results seen for the BvM classifier, a detailed analysis of features showed various geometrical and morphometrical features guiding the automated detection of malignant subtypes. There was a distinct variation between nuclear haematoxylin OD, cytoplasmic and eosin OD, and nucleus/cell ratio (*p* < 0.01) between the different malignant tumours. Interestingly, no measurable difference was seen between nuclear circularity and eccentricity for this task (Fig. 5a). For the TG classifier, the analysis showed that there was a meaningful variation between nuclear circularity, cytoplasmic eosin OD, and nucleus/cell ratio (*p* < 0.01) between low and high-grade tumours (Fig. 6a).

### Quantitative morphometrical feature analysis

#### Cellularity

The average number of cells in malignant tumours was higher than benign tumours (average cellularity per case calculated across five standardised ROIs). Cellularity varied considerably between the groups (*p* < 0.01) (Fig. 4b). Comparison of malignant tumours showed the highest cellularity in Ca ex PA, followed by AdCC and ACC. MEC demonstrated lower cellularity compared to other SGT. The analysis demonstrated a distinct variation in cellularity across the four SGT groups(*p* < 0.01) (Fig. 5b). The average number of cells in high-grade tumours was also notably higher than low-grade tumours (*p* < 0.01) (Fig. 6b).

#### Spatial analysis

**Fig. 7:**
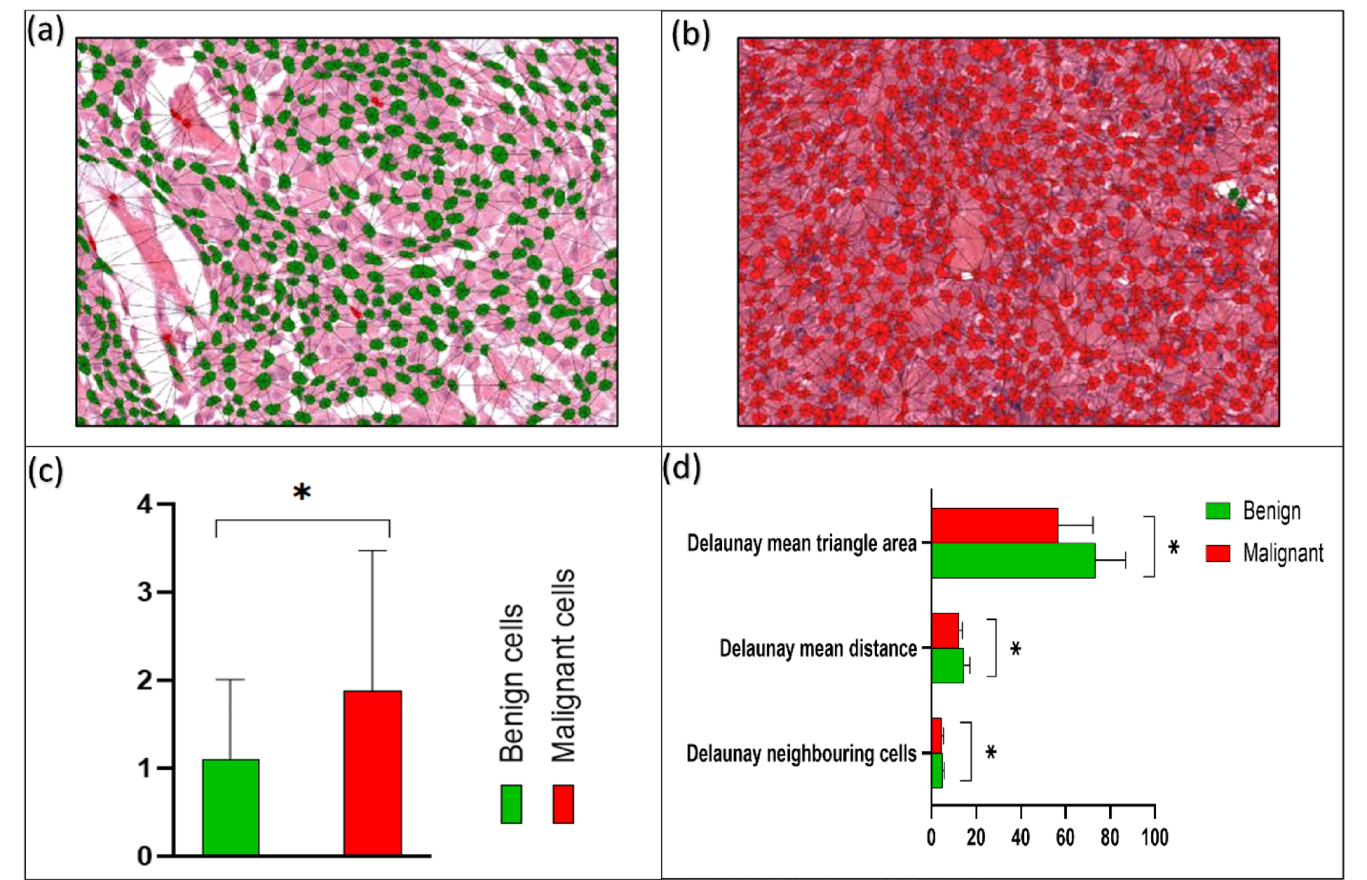
Spatial and proximity analysis of benign vs. malignant tumours (test *n* = 80). (**a**,**b**) Showing spatial orientation networks of benign (**a**) and malignant (**b**) cells (magnification ×20). (**c**) Average centroid distance between benign and malignant cells (µm). (**d**) Cluster spatial analysis of Delaunay features for benign and malignant tumours. Error bars = standard deviation. *p < 0.01 (T-Test (two-tailed).

The average centroid distance showed a notable difference between cells in malignant and benign SGT, with a smaller distance seen in malignant SGT (*p* < 0.01) in keeping with increased cellularity (Fig. 7a–c). Cluster spatial analysis showed a pronounced difference in Delaunay neighbouring cells in benign and malignant SGT (*p* < 0.01). Furthermore, there was a distinct variation between the Delaunay mean intercellular distance between benign and malignant tumours (*p* < 0.01). In addition, the Delaunay mean triangle area was also different between benign and malignant SGT (*p* < 0.01) (Fig. 7d).

**Fig. 8:**
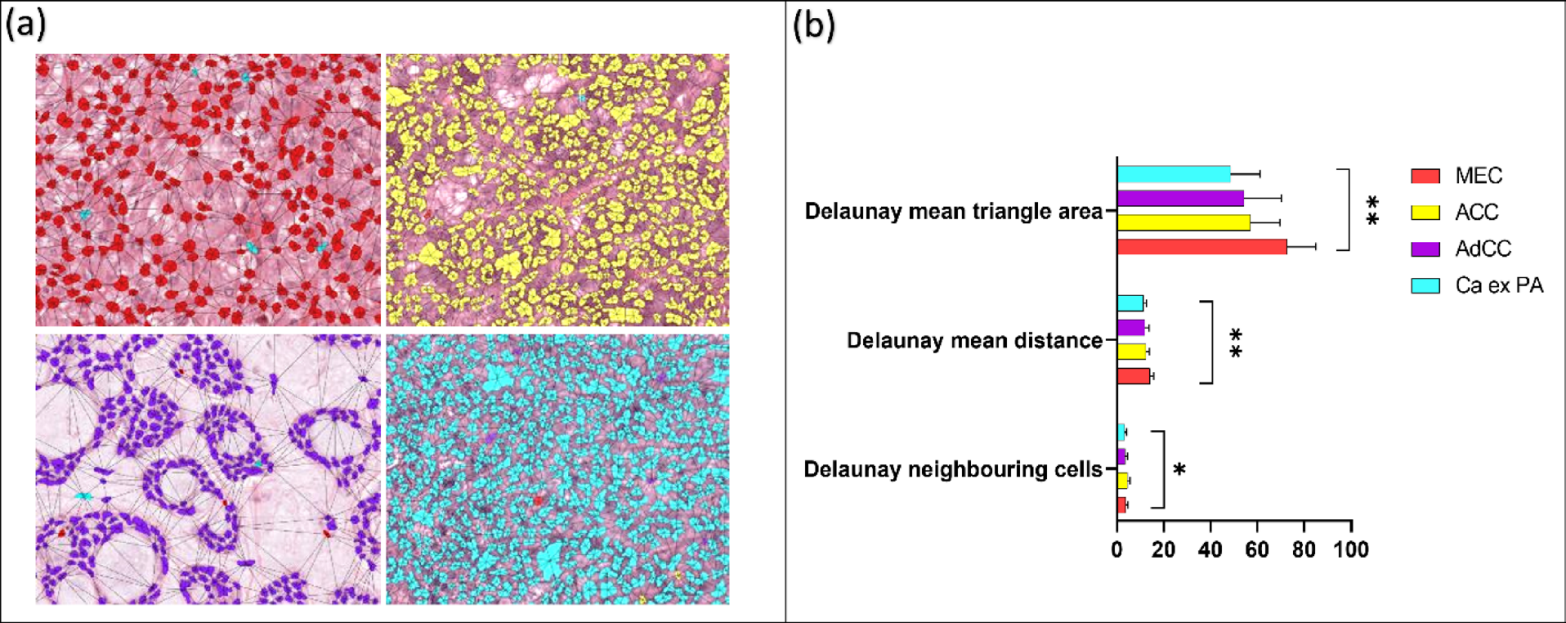
Spatial analysis of malignant subtypes (test *n* = 40). (**a**) Showing spatial orientation networks of different malignant subtypes (blue-Ca ex PA, purple-AdCC, yellow-ACC, red-MEC) (magnification ×20). (**b**) Cluster spatial analysis of Delaunay features for malignant subtypes. Error bars = standard deviation. *p < 0.05, **p < 0.01 (multiple comparison one-way ANOVA).

Spatial analysis for different malignant subtypes showed the highest number of Delaunay neighbouring cells in ACC compared to MEC, AdCC and Ca-ex-PA (*p* < 0.05) (Fig. 8a,b). The Delaunay mean distance was also showed pronounced difference between different types of malignant SGT (*p* < 0.01). In addition, the Delaunay mean triangle area also showed a notable difference (*p* < 0.01) between subtypes with the highest area seen in MEC and the lowest in AdCC (Fig. 8b).

**Fig. 9:**
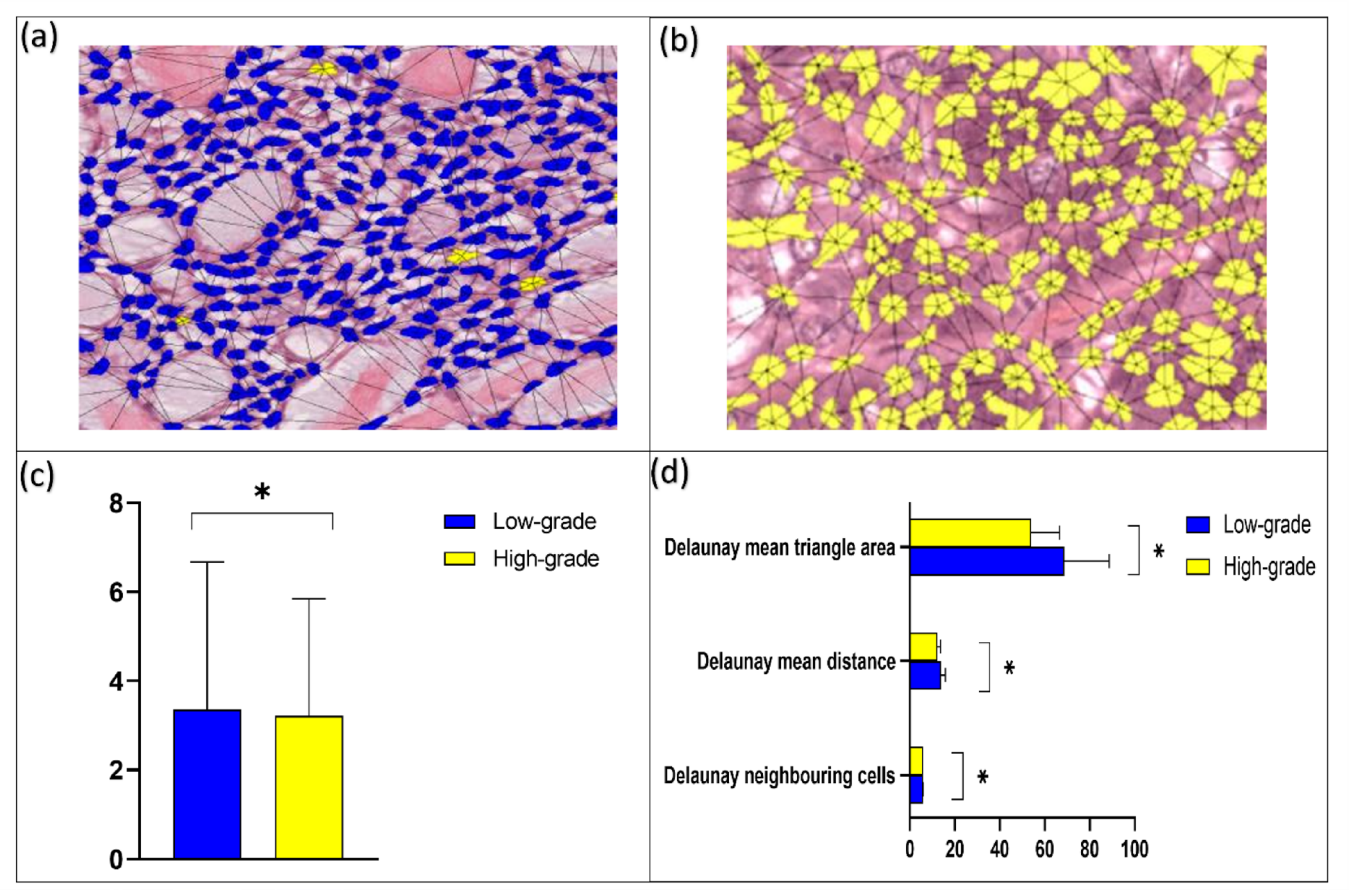
Spatial and proximity analysis of low- and high-grade tumours (test *n* = 40). (**a**,**b**) Showing spatial orientation networks of low-grade AdCC (**a**) and high-grade MEC (**b**) cells (magnification ×20). (**c**) Average centroid distance between low and high-grade tumours (µm). (**d**) Cluster spatial analysis of Delaunay features for low and high-grade tumours. Error bars = standard deviation. *p < 0.01(T-Test two-tailed).

Dynamic interaction between low and high-grade cells creates topographical features that explain spatial orientation (Fig. 9‎a,b). The average centroid distance showed a distinct variation between cells in low and high-grade tumours, with a smaller distance seen in high-grade tumours (*p* < 0.01) (Fig. 9c). Cluster spatial analysis showed a notable difference in the number of neighbouring cells in low and high-grade tumours (*p* < 0.01). Furthermore, there was a measurable difference between the Delaunay mean intercellular distance between low- and high-grade tumours (*p* < 0.01). In addition, the Delaunay mean triangle area also varied between low- and high-grades tumours (*p* < 0.01) (Fig. 9d).

## Discussion

Computational pathology (ML, DL, and AI) has been shown to provide objective and accurate quantification and classification in a range of cancers^5–9,25−31^. This study presents a novel application of AI, integrating ML and DL, for the histological analysis of SGT using H&E-stained WSIs. Our approach uniquely addresses the full diagnostic spectrum of benign versus malignant classification, malignant subtyping, and preliminary tumour grading within a real-world dataset. While earlier studies have demonstrated the feasibility of AI in SGT classification^15,16^, these efforts primarily focused on subtype identification using large datasets. In contrast, our model incorporates interpretable, nuclear and cellular level features to support a more comprehensive diagnostic workflow.

Our ML benign vs. malignant (BvM) classifier has achieved an excellent accuracy with F1 and AUROC values of 0.95. Although prior work also has reported tumour detection on gross specimens using hyperspectral imaging^32^, direct comparisons remain limited due to differences in imaging modality and diagnostic focus. Therefore, a direct comparison of our findings with relevant studies is not possible. Nonetheless, comparable concepts have been explored in a number of studies; for example, one study in breast tissue attempted to distinguish between non-carcinoma (normal and benign) and carcinoma (in situ and invasive) and reached an accuracy of 83.3%^25^. Another study of benign and malignant breast lesions using a class structure-based deep learning model showed 93% accuracy^33^. In prostate adenocarcinoma, an AUC of 0.98 and 0.99, respectively, have been reached using DL to distinguish between benign and malignant tumours on large datasets^27,31^. Although we have shown novel and interesting data using ML classifiers and DL models for SGT analysis, it is apparent that modern DL techniques can offer significantly higher performance and accuracy at a WSI level on larger datasets, and we are already exploring this as an extension to the existing work. However, SGT are much rarer compared to breast, prostate, and other adenocarcinomas; therefore, curating large multicentre datasets (similar to published studies with large scale validation) is extremely challenging.

Malignant SGT subtyping can require the use of several further IHC stains and referral to specialist pathologists with associated time and cost implications as well as variation in the availability of these tests, in particular in developing countries. Due to time and data restraints, only four common types of malignant SGT could be included for comparison; these tumours were selected as they account for the majority of malignant SGT in reported studies^34,35^. Our MST classifier showed excellent accuracy (F1 scores- 0.95, AUROC- 0.97). Interestingly, but not surprisingly, the performance was inferior for DL for this task. DL algorithms require larger datasets, especially for challenging tasks, and it is likely that the use of ROIs and four different classes/tumours in a relatively small dataset was insufficient to achieve good accuracy. Recent advancements in DL, including transformer-based architectures have demonstrated excellent performance in classifying SGT from large-scale datasets^15^. However, despite high accuracy in subtype classification, most existing models have focused on categorical prediction without exploring nuclear feature analysis. In addition, DL has been used to subtype other glandular carcinomas, such as lung Adenocarcinoma (LUAD) vs. squamous cell carcinoma (LUSC) or normal lung tissue in much larger datasets at WSI level (AUC of 0.97)^36^.

Despite evidence suggesting that grading systems produce clinically useful information, all grading schemes are arbitrary and open to interpretation. In addition, grading in these systems is often subjective and dependent on pathologist experience^37,38^. In AdCC, the 2022 WHO classification highlights the presence of any solid tumour component as a key indicator of high-grade transformation, in line with prior evidence^39^. In our cohort, high-grade AdCC cases identified by the AI model also demonstrated necrosis, a feature widely recognised as a reproducible marker of aggressive behaviour. Nonetheless, we acknowledge that histological grading in salivary gland tumours remains challenging due to biological variability, particularly in mucoepidermoid carcinoma (MEC), where certain oncocytic variants may appear solid but behave indolently. The grading component of this study was therefore designed as an exploratory proof-of-concept. Our aim was not to replace current grading systems but to evaluate the potential of AI in recognizing morphological correlates of grade. Ki-67 immunostaining is used frequently to assess tumour behaviour and has been shown to significantly correlate with histological grade in MEC, but no such correlation was observed in AdCC^40^. In general, the association between IHC markers and histological grade or clinical outcomes in SGT is poorly understood^41^. Furthermore, Interobserver variability in SGT grading can lead to diagnostic inconsistencies and may affect the reliability of AI training data. However, AI has the potential to apply grading criteria consistently, reducing such variability when trained on expert consensus annotations. Future studies should incorporate larger datasets and outcome-based validation to support more reproducible grading models.

Our TG-tumour grading ML classifier showed an excellent performance with an F1-score of 0.87. In comparison, the best performing DL algorithm was EfficientNet-B0 with an inferior F1-score. Nonetheless, the result was comparable to previously reported work in breast cancer grading^42^.

Although ML offers the advantage of automated analysis of histopathological features, it requires a large amount of training and annotations, making it tedious and time-consuming. DL algorithms can learn directly from raw data, but it can be difficult to establish how algorithms make the decision from input data and arrive at a prediction^43^. In addition, DL algorithms are data-hungry, often requiring large cohorts. Our study uses a hybrid approach for SGT classification and segmentation, including analysis of morphological, geometrical and colour features that ML uses for decision making and comparing that with DL algorithms (using ML generated ROI level features).

For benign vs. malignant SGT, the analysis of the geometrical and morphometrical features demonstrated a notable difference between nuclear circularity, nuclear haematoxylin, cytoplasmic eosin and nucleus/cell ratio. This is biologically relevant as malignant cells are known to have a higher and abnormal DNA content (i.e., hyperchromatism), leading to a higher nuclear haematoxylin staining and nuclear/cell ratio^44^. This also highlights potential for application and translation to rapid diagnostic screening methods such as diffquik fine needle aspiration cytology. Nuclear haematoxylin staining and nucleus/cell ratio were also statistically different between different malignant SGT, which could be related to the degree of differentiation or histological grade of those tumours. Also, similar findings were seen in tumour grading classifier, which makes biological relevance as higher grade and poorly differentiated neoplasms exhibit less circularity and more pleomorphism. In addition, high-grade tumours showed increased nuclear cellular area ratio, which is a well-known feature of malignant and high-grade tumours^44–46^.

Assessment of tumour cellularity is a subjective and tedious process, not routinely used for diagnosis or prognosis prediction, and with concerns of inter-observer variability among pathologists. ML and DL can aid this process and have been shown to objectively quantify tumour cellularity^47^. Our results show higher cellularity in malignant and high grades tumours compared to benign and low grades. This observation is in agreement with the literature as malignant and high grade tumours are known to be more cellular, but this quantification is rarely done in practice, in particular for SGT^44,46,48^.

Our paper is also the first to report the importance of spatial characteristics in SGT. This is important as the tumour microenvironment has been shown to play a key role in progression and prognosis of numerous neoplasms. Furthermore, in many types of cancers, histopathological analysis can show cells growing in clusters and show architectural patterns and organisation. Ali et al., (2013) reported a cell cluster graph (CCG) which computationally characterised prostate cancer tissue images according to spatial distribution and correlated it with histological grading^49^. Our study shows that spatial orientation and clustering of cells are different between benign and malignant tumours, different malignant subtypes as well as low and high grades tumours and this might be a valuable adjunct for differentiating between them.

AI-based classifiers such as those presented in this study could be integrated into diagnostic workflows as decision-support tools. One potential application is as a screening system to flag potentially malignant or high-grade tumours for prioritized review by pathologists. Additionally, these models could assist in providing an objective judgment in cases of tumour grading, where subjective interpretation often contributes to interobserver variability. This functionality may be particularly valuable in diagnostically complex entities, or in institutions with limited access to expert subspecialty consultation. Nonetheless, clinical adoption would require prospective validation, interpretability of predictions, and compliance with regulatory and ethical standards.

Despite the novelty of our findings, our training dataset included cases from a single centre. However, this is a centre of excellence with regards to SGT analysis, receiving routine and referral cases from not only across the region but also nationally and internationally, which would mitigate bias and add to the robustness of the trained algorithms. Although the sample size is relatively limited compared to large-scale AI studies, it reflects the real-world distribution and rarity of salivary gland tumours; thus, this work serves as a feasibility study demonstrating the potential of AI-driven models in supporting histopathological classification and grading within this uncommon tumour group. Furthermore, underrepresentation of rare salivary gland tumour subtypes, which were too infrequent for reliable model training or evaluation. This restricts the model’s generalizability across the full spectrum of salivary gland neoplasms. Future scalability will depend on larger, multicentre datasets and approaches such as data augmentation or transfer learning.

To conclude, AI has an enormous potential to aid diagnosis and improve patient care. Our novel findings show that AI can be used for analysis, quantification, and differentiation between salivary gland tumours. A larger multicentre cohort needs to be analysed to determine the true significance and clinical usefulness of these findings.

## Data Availability

The data that support the findings of this study are available from the corresponding author upon reasonable request. All ML classifiers (BvM, MST and TG) are available at https://github.com/IbrahimSalsanie.
